# Burden of Ischemic Heart Disease Attributable to Low Omega-3 Fatty Acids Intake in Iran: Findings from the Global Burden of Disease Study 2010

**Published:** 2016-01-13

**Authors:** Sara Nejatinamini, Asal Ataie-Jafari, Anoosheh Ghasemian, Roya Kelishadi, Alireza Khajavi, Amir Kasaeian, Shirin Djalalinia, Fahad Saqib, Somayye Majidi, Roxana Abdolmaleki, Mehrnaz Hosseini, Hamid Asayesh, Mostafa Qorbani

**Affiliations:** 1 *Non-Communicable Diseases Research Center, Endocrinology and Metabolism Population Sciences Institute, Tehran University of Medical Sciences, Tehran, Iran.*; 2 *Department of Nutrition, Science and Research Branch, Islamic Azad University, Tehran, Iran.*; 3 *Child Growth and Development Research Center, Research Institute for Primordial Prevention of Non-Communicable Disease, Isfahan University of Medical Sciences, Isfahan, Iran.*; 4 *Hematology-Oncology and Stem Cell Transplantation Research Center, Tehran University of Medical Sciences, Tehran, Iran.*; 5 *Department of Medical Emergencies, Qom University of Medical Sciences, Qom, Iran.*; 6 *Department of Community Medicine, School of Medicine, Alborz University of Medical Sciences, Karaj, Iran.*

**Keywords:** Cardiovascular diseases, Myocardial ischemia, Fatty acid, omega-3, Cost of illness, Iran

## Abstract

**Background: **Dietary risk factors constitute some of the leading risk factors for cardiovascular disease in Iran. The current study reports the burden of ischemic heart disease (IHD) attributable to a low omega-3 fatty acids intake in Iran using the data of the Global Burden of Disease (GBD) Study 2010.

**Methods: **We used data on Iran for the years 1990, 2005, and 2010 derived from the GBD Study conducted by the Institute for Health Metrics and Evaluation (IHME) in 2010. Using the comparative risk assessment, we calculated the proportion of death, years of life lost, years lived with disability, and disability-adjusted life years (DALYs) caused by IHD attributable to a low omega-3 fatty acids intake in the GBD studies from 1990 to 2010.

**Results: **In 1990, a dietary pattern low in seafood omega-3 fatty acids intake was responsible for 423 (95% uncertainty interval [UI], 300 to 559), 3000 (95% UI, 2182 to 3840), and 4743 (95% UI, 3280 to 6047) DALYs per 100000 persons in the age groups of 15 to 49 years, 50 to 69 years, and 70+ years — respectively — in both sexes. The DALY rates decreased to 250 (95% UI, 172 to 331), 2078 (95% UI, 1446 to 2729), and 3911 (95% UI, 2736 to 5142) in 2010. The death rates per 100000 persons in the mentioned age groups were 9 (95% UI, 6 to 12), 113 (95% UI, 82 to 144), and 366 (95% UI, 255 to 469) in 1990 versus 6 (95% UI, 4 to 7), 76 (95% UI, 53 to 99), and 344 (95% UI, 241 to 453) in 2010. The burden of IHD attributable to diet low in seafood omega-3 was 1.3% (95% UI, 0.97 to 1.7) of the total DALYs in 1990 and 2.0% (95% UI, 1.45 to 2.63) in 2010 for Iran.

**Conclusion**: The findings of the GBD Study 2010 showed a declining trend in the burden of IHD attributable to a low omega-3 fatty acids intake in a period of 20 years. Additional disease burden studies at national and sub-national levels in Iran using more data sources are suggested for public health priorities and planning public health strategies.

## Introduction

Noncommunicable diseases (NCDs) are the leading cause of global mortality accounting for 65.46% of deaths worldwide. Among them, ischemic heart disease (IHD) is the most common cause of death in the world. Globally and Iran, IHD accounts for 13.32% and 25.43% of all deaths respectively.^1^ Looking beyond mortality, the burden of disease attributed to cardiovascular disease is also considerably high. IHD also results in substantial long-term morbidity and is the leading cause of the overall disease burden, as measured in disability-adjusted life years (DALYs) lost.^2^ Iran possibly has a higher burden than do other countries in the Eastern Mediterranean region.^3^ Thus, there is a special and urgent need for data and treatment strategies apropos IHD in Iran.

Addressing risk factors such as tobacco use, unhealthy diet, obesity, physical inactivity, high blood pressure, diabetes, and raised lipids can prevent most cardiovascular diseases. Overall, the leading risk factor accounting for the most disease burden as well as IHD in Iran is dietary risk.^1^ Several dietary factors affect IHD. One of the most important ones is the consumption of fruits, vegetables, nuts and seeds, whole grains, processed meat, polyunsaturated fats, and seafood omega-3 fatty acids.^4^^-^^12^ Seafood omega-3 fatty acids consumption is related to IHD risk reduction in both observational and clinical intervention trials and the 2010 Dietary Guideline for Americans recommends the consumption of 8 ounces or more of seafood weekly to provide an average consumption of 1,750 mg/wk (250 mg/d) of eicosapentaenoic acid (EPA; 20:5n-3) and docosahexaenoic acid (DHA; 22:6n-3) (United States Department of Agriculture; Department of Health and Human Services. Dietary Guidelines for Americans, 2010; US Government Printing Office: Washington, DC, U.S.A., 2010).

Burden of disease analysis provides a unique perspective on health. A comparative assessment of the contribution of risk factors to disease burden and mortality is useful for health policy with the aim of reducing disease burden, promoting health, and determining priority actions and interventions in response to this crisis. The current study, therefore, aims to report the burden of IHD attributable to a low omega-3 fatty acids intake in Iran using the data provided by the Global Burden of Disease (GBD) Study 2010, which was conducted by the Institute for Health Metrics and Evaluation (IHME). 

## Methods

The GBD Study 2010 calculated the proportion of death and DALYs attributable to a low omega-3 fatty acids intake between 1990 and 2010. The details of data, data quality, and statistical models for the GBD Study 2010 estimation have been described previously.^13^^-^^20^ In brief, using the comparative risk assessment approach and keeping other independent factors, the proportion of IHD burden or deaths caused by a low intake of omega-3 fatty acids was estimated. A low intake of seafood omega-3 fatty acids was defined as a dietary intake of EPA and DHA < 250 mg/d.^8^ In the first stage, risk-outcome pairs were defined according to the criteria for causal associations.^20^ Next, the distribution of exposure to each risk factor was determined. The main data sources for this estimation were published data and unpublished data extracted from a systematic search. Statistical models such as space-time/Gaussian process regression model or meta-regression were also designed to produce a complete dataset of exposure distribution. Then, etiological effect size, often relative risk per unit of exposure for each risk-outcome pair, was calculated and theoretical minimum risk exposure distribution was included. In the final stage, death and disease burden attributable to risk factors were estimated by comparing the current exposure distribution to the theoretical minimum risk exposure distribution. Uncertainty in the estimates was also calculated. Eventually, the exposure estimate, relative risk, theoretical minimum risk distribution, and uncertainty in the first outcome rates were propagated into the last estimate.

In the present study, for the first time in Iran, we provide a summary of the burden of IHD attributable to a low omega-3 fatty acids intake. To that end, we extracted the required data in terms of sex and age groups from the website of the IHME. We analyzed and reformulated the data, drew new graphs, and critiqued the results. These data were DALY and death rates per 100 000 persons (95% uncertainty interval [UI]) caused by IHD attributable to a low omega-3 fatty acids intake in Iran in the years 1990, 1995, 2000, 2005, and 2010. In addition, we extracted data on age-standardized rates and data on all ages and calculated the percentages of change in deaths and DALYs between 1990 and 2010. We depicted the time trends of the DALY and death rates of IHD attributable to a low omega-3 fatty acids intake by age groups and by sex from 1990 to 2010 in graphs and prepared the figures using R software for Windows. The entire measures are reported with a 95% UI.

## Results

The DALY and death rates per 100 000 persons caused by IHD attributable to a low omega-3 fatty acids intake from 1990 to 2010 are presented in Table 1 and Table 2. In 1990, a dietary pattern low in seafood omega-3 fatty acids intake was responsible for 423 (95% UI, 300 to 559), 3000 (95% UI, 2 182 to 3840), and 4743 (95% UI, 3280 to 6047) DALYs per 100000 persons in the age group of 15 to 49 years, 50 to 69 years, and 70+ years - respectively - in both sexes. The DALY rates decreased to 250 (95% UI, 172 to 331), 2078 (95% UI, 1 446 to 2729), and 3 911 (95% UI, 2736 to 5142) in 2010. The death rates per 100 000 persons in the mentioned age groups were 9 (95% UI, 6 to 12), 113 (95% UI, 82 to 144), and 366 (95% UI, 255 to 469) in 1990 versus 6 (95% UI, 4 to 7), 76 (95% UI, 53 to 99), and 344 (95% UI, 241 to 453) in 2010.

**Figure 1:**
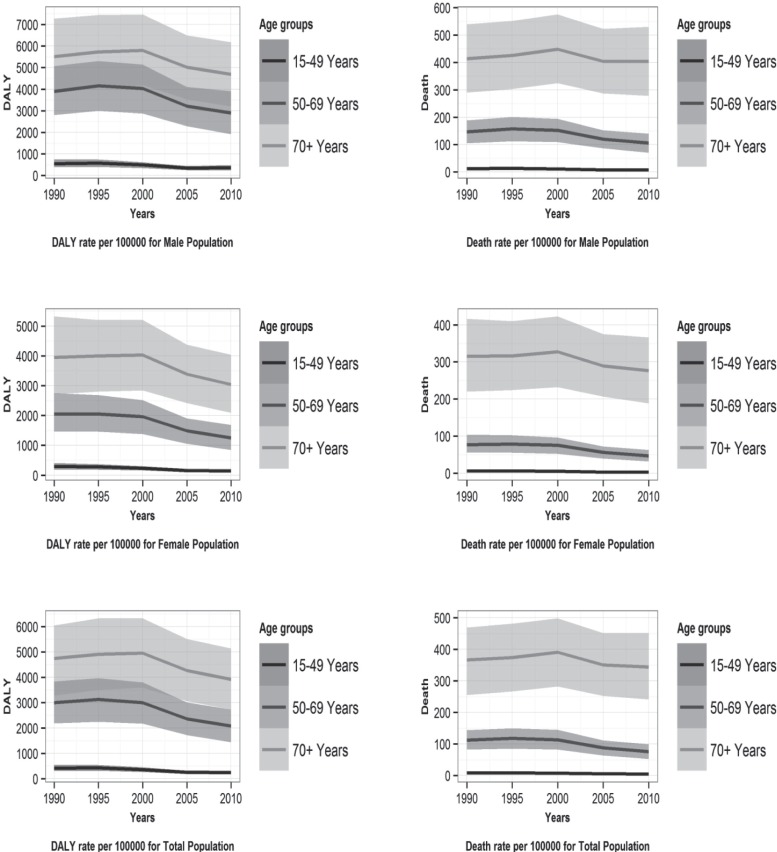
Time trends of disability-adjusted life year (DALY) and deaths rates of ischemic heart disease attributable to low omega-3 fatty acids intake by age groups for men, women, and both sexes from 1990 to 2010 in Iran

**Figure 2:**
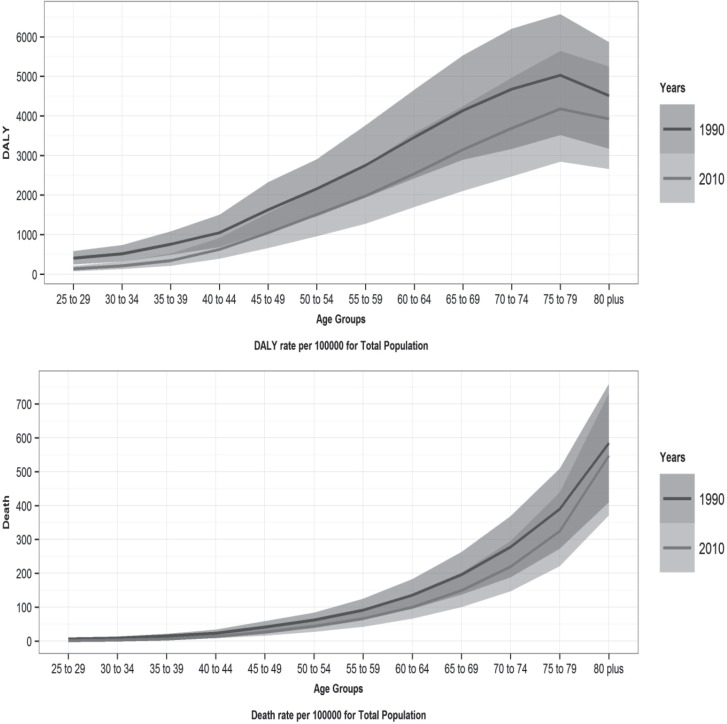
Trends of disability-adjusted life year (DALY) and death rates of ischemic heart disease attributable to low omega-3 fatty acids intake by age groups in1990 and 2010 in Iran

Figure 1 illustrates the time trends of the DALY and death rates caused by IHD attributable to a low omega-3 fatty acids intake by age groups for men, women, and both sexes from 1990 to 2010 in Iran. Over this period, a low intake of omega-3 fatty acids caused the most burden and deaths in the age group of 70+ years compared to the other age groups in men, women, and both sexes. The DALY and death rates caused by IHD attributable to a low omega-3 fatty acids intake by age groups in 1990 and 2010 in Iran are depicted in Figure 2. It demonstrates that the attributed DALYs and deaths increased with age and reached the highest level in the oldest age group.

In women, the age-standardized DALY rates declined by 38.1% between 1990 and 2010, but the decrease was lower (25.7%) for men. The female and male age-standardized mortality rates also decreased by 28.5% and 19.6%, respectively.

The burden of IHD attributable to diet low in seafood omega-3 was 1.3% (95% UI, 0.97 to 1.7) of the total DALYs in 1990 and 2.0% (95% UI, 1.45 to 2.63) in 2010 for Iran; thus, its share of disease burden demonstrated a rise. Table 3 compares the total DALY rates per 100 000 persons attributable to diet low in seafood omega-3 fatty acids by sex, for all ages, in the world, Eastern Mediterranean region (Egypt and Turkey), and Iran in 1990 and 2010. In these three countries, Iran and Turkey had the same trend of total DALY rates per 100 000 persons during this period, which was also similar to the worldwide trend. However, Egypt by comparison with the other two countries showed an increasing pattern in the total DALY rates per 100 000 persons attributable to diet low in seafood omega-3 fatty acids from 1990 to 2010.

**Table 1:** Disability-adjusted life year (DALY) rates per 100 000 persons (95% uncertainty interval) of ischemic heart disease attributable to low omega-3 fatty acids intake from 1990 to 2010 in Iran

	DALY rates per 100 000 persons (95% uncertainty interval)					%Δ (1990-2010)

	1990	1995	2000	2005	2010	
Both Sexes						
15-49 y	423 (300-559)	430 (313-550)	365 (266-458)	252 (185-320)	250 (172-331)	-40.8
50-69 y	3000 (2182-3840)	3124 (2247-3961)	2997 (2175-3804)	2358 (1716-3008)	2078 (1446-2729)	-30.7
70+ y	4743 (3280-6047)	4908 (3494-6322)	4956 (3596-6320)	4265 (3059-5503)	3911 (2736-5142)	-17.5
Age-standardized	1033 (740-1320)	1059 (767-1347)	1007 (733-1270)	795 (580-1007)	719 (512-924)	-30.3
All ages	540 (388-693)	579 (420-737)	594 (432-749)	528 (386-668)	538 (385-695)	-0.37
						
Male						
15-49 y	552 (387-743)	584 (407-748)	495 (348-624)	346 (246-441)	356 (233-481)	-35.5
50-69 y	3894 (2809-5074)	4165 (2998-5306)	4034 (2871-5139)	3223 (2294-4111)	2909 (1915-3913)	-25.2
70+ y	5507 (3867-7281)	5728 (4047-7434)	5798 (4193-7466)	5024 (3538-6499)	4690 (3201-6188)	-14.8
Age-standardized	1306 (939-1688)	1379 (987-1755)	1320 (945-1668)	1043 (752-1324)	970 (671-1263)	-25.7
All ages	694 (498-899)	762 (545-968)	778 (557-981)	699 (505-886)	730 (504-947)	5.1
						
Female						
15-49 y	298 (196-411)	279 (189-374)	236 (167-305)	156 (110-212)	142 (92-202)	-52.3
50-69 y	2047 (1457-2751)	2050 (1460-2686)	1966 (1376-2521)	1490 (1049-1902)	1250 (844-1690)	-38.9
70+ y	3944 (2712-5322)	4003 (2805-5204)	4030 (2845-5207)	3383 (2420-4373)	3036 (2096-4041)	-23.0
Age-standardized	750 (545-980)	734 (523-949)	696 (498-888)	538 (391-683)	464 (331-613)	-38.1
All ages	382 (278-501)	393 (280-509)	407 (293-519)	351 (446-256)	341 (242-447)	-10.7

%Δ , Percent changes between 1990 and 2010

**Table 2:** Death rates per 100 000 persons (95% uncertainty interval) of ischemic heart disease attributable to low omega-3 fatty acids intake from 1990 to 2010 in Iran

	Death rates per 100 000 persons (95% uncertainty interval)					%Δ (1990-2010)

	1990	1995	2000	2005	2010	
Both Sexes						
15-49 y	9 (6-12)	9 (7-12)	8 (6-10)	6 (4-7)	6 (4-7)	-33.3
50-69 y	113 (82-144)	119 (86-150)	114 (83-145)	88 (64-112)	76 (53-99)	-32.7
70+ y	366 (255-469)	374 (267-481)	390 (283-498)	351 (252-452)	344 (241-453)	-6.0
Age-standardized	46 (33-58)	47 (34-61)	46 (34-59)	38 (28-48)	35 (25-46)	-23.9
All ages	21 (15-26)	23 (17-29)	25 (18-32)	24 (17-30)	25 (18-32)	19.0
						
Male						
15-49 y	12 (8-16)	13 (9-16)	11 (8-14)	8 (6-10)	8 (5-11)	-33.3
50-69 y	146 (105-189)	157 (113-201)	152 (109-194)	120 (86-152)	106 (70-141)	-27.3
70+ y	414 (290-540)	426 (303-551)	448 (324-575)	404 (287-522)	404 (278-530)	-2.4
Age-standardized	56 (40-72)	59 (42-75)	58 (41-74)	48 (34-61)	45 (31-59)	-19.6
All ages	26 (19-33)	29 (21-37)	32 (23-40)	31 (22 39)	32 (22-42)	23.0
						
Female						
15-49 y	6 (4-9)	6 (4-8)	5 (4-9)	3 (2-5)	3 (2-4)	-50
50-69 y	77 (55-104)	78 (56-103)	75 (52-96)	56 (40-72)	47 (31-63)	-38.9
70+ y	316 (220-416)	316 (224-410)	327 (232-423)	289 (207-375)	276 (188-366)	-20.2
Age-standardized	35 (26-46)	36 (26-46)	35 (25-44)	28 (20-36)	25 (17-33)	-28.5
All ages	15 (11-20)	16 (12-21)	18 (13-23)	17 (12-22)	17 (12-22)	13.3

%Δ , Percent changes between 1990 and 2010

**Table 3:** Total disability-adjusted life year (DALY) rates per 100 000 persons attributable to diet low in seafood omega-3 fatty acids by sex, for all ages, in the world, Eastern Mediterranean region (Turkey and Egypt), and Iran in 1990 and 2010

	Men		Women		Both Sexes	
	1990	2010	1990	2010	1990	2010
World	698 (507-889)	583 (423-740)	366 (266-468)	283 (207-360)	527 (384-668)	430 (314-548)
Turkey	1560 (1141-1975)	968 (695-1233)	732 (536-934)	439 (320-567)	1137 (835-1436)	694 (503-882)
Egypt	982 (691-1334)	1125 (801-1409)	596 (427-858)	624 (450-797)	782 (559-1038)	867 (627-1091)
Iran	1306 (939-1688)	970 (671-1263)	750 (545-980)	464 (331-613)	1033 (740-1320)	719 (512-924)

## Discussion

The current study presents the burden of IHD attributable to diet low in seafood omega-3 fatty acids in Iran from the results of the GBD Study 2010. The mortality and disability caused by IHD attributable to a low omega-3 fatty acids intake in almost each age group in Iran has exhibited a drop in the past two decades. 

Fish is the major food source of long-chain n-3 polyunsaturated fatty acid (LCn-3 PUFA), including EPA (20:5n-3) and DHA (22:6n-3). Its per capita consumption was 1 kg/y in 1980 and reached 7.6 kg in 2009 (FAO publications related to aquaculture for Iran. Data sources from: Planning and budget directorate of Iranian Fisheries Org 2011). The expansion of aquaculture throughout the country, together with an increase in people's knowledge about fish as a healthy food, has helped change the general population’s attitude toward fish and marketing campaigns by Iranian fisheries. Moreover, the Iranian Ministry of Health and Medical Education has also sought to encourage fish consumption in Iran. Per capita seafood consumption varies widely by region and country: The global average is about 18.8 kg live-weight equivalent, with Japan having the highest rank reaching over 58 kg (Fisheries and Aquaculture Department (FAO) The State of the World Fisheries and Aquaculture 2012. FAO; Rome, Italy: 2012. p. 209). 

Several prospective epidemiological studies have reported that men consuming at least some fish weekly have a lower IHD mortality rate than do men eating none.^21^^-^^24^ Cardiovascular disease is a severe burden on the health care system in Iran (Ahmadvand AR, Ahmadi A, Ardalan A, Eskandarizade A, Esmaelnasab N, Osoli M. Epidemiology textbook of prevalence disease in Iran. Ischemic Heart Diseases. Vol (1). Tehran: 2014;3:55-60). The prevention of such diseases is a public health goal and comprises several strategies, of which one of the most effective may be the inclusion of fish in the diet.^25^ There are some critical concerns regarding fish consumption in Iran - including the concentration of n-3 fatty acids in local fish species, residues of some unwanted chemicals (e.g., methylmercury, pesticides, and fertilizers), improper methods of fish storage, lack of habitual fish consumption in some parts of the country especially among children (issues of culture and palatability), and improper fish preparation and cooking at household level (dominated by the prolonged frying method). However, the factors that can act as key determinants of fish consumption in the general population include fish supply (physical access) in terms of production, importation, and processing capacities together with the eventual impact on the prices (economic access).

The global rate of DALYs per 100 000 persons attributable to a low omega-3 fatty acids intake has decreased from 527 (95% UI, 668 to 384) to 430 (95% UI, 548 to 314) over the past 2 decades. The world consumption of fish has risen during the recent years, increasing not only the rates of proteins and calories (11% to 24%), with 96% of easiness in digestion, but also the rates of unsaturated fatty acids such as omega-3. Moreover, despite the increase in the world’s population, living standards have improved.^26^

To bring people closer to meeting the goal of consuming fatty fish, intensive intervention is needed at three steps: first, to shift the non-fish eaters to the adoption of regular fish consumption; second, to more than double the amount of fish consumed; and third, to move consumers to dramatically increase the amount of fatty fish consumed.

For the time being, there is a consumption rate of 7.35 kg fish per capita in Iran (FAO publications related to aquaculture for Iran. Data sources from: Planning and budget directorate of Iranian Fisheries Org 2011). Considering the figure of 16.5 kg fish per capita in the world, a big difference exists between Iran and other countries. Fish is not a popular part of the Iranian diet, especially in its central cities which constitute the main proportion of the entire population. In other words, despite huge achievements in the fisheries sector, there is still a long way to go before the nutrition policy goal is achieved. Modulation of the national habitual consumption of fish will need careful examination of evidence for the effectiveness of various policy options such as voluntary agreements, subsidies, taxes, marketing, and information campaigns. 

In Iran, the age-standardized rates for IHD decreased between 1990 and 2010, although stroke rates were relatively constant. Rapid reductions in fertility and age-speciﬁc mortality rates have led to many more Iranian individuals living to an old age, when rates of chronic disability are high. The massive declines in the rates of death from IHD in many high-income countries have also been attributed to improved management of acute cardiac events and post-event care.

The burden of IHD attributable to a low intake of omega-3 was slightly higher in men than in women: approximately 2.5% of total DALYs versus 1.5%. The existence of gender differences in cardiovascular disease following LCn-3 PUFA consumption suggests that sex hormones play a role in cardio-protection. It is well established that hormone-dependent gender differences exist in vascular function. Estrogen causes vasodilatation, affects blood pressure, inhibits the response of blood vessels to injury, and retards the development of atherosclerosis.^27^ In addition, lipid abnormalities contribute substantially to atherosclerosis and are also regulated by sex steroid hormones, principally by way of hepatic lipoprotein metabolism.^28^ Gender differences in LCn-3 PUFA metabolism have also recently been reported. In a large population study, women had lower proportions of EPA and DPA in phospholipids and a higher proportion of DHA, though the intake of fish fat did not differ in terms of gender.^29^

The present study has important limitations similar to those in the GBD Study, which are explained elsewhere.^13^^-^^20^ One of the main restrictions concerns the data sources, which affected the exposure estimations for risk factors such as low omega-3 fatty acids in Iran. Population-based epidemiological studies and national and sub-national health surveys were the sources of data, which were ignored for the estimations in Iran. Due to the lack of accessibility to data sources, there was a limitation for the accurate estimates, reported by uncertainties. Because of the incompleteness of data, statistical models were used to provide a complete dataset of exposure distribution. The GBD estimation is robust at global and conservatively at national level. What is more, the consumption of see foods as the main source of the intake of omega-3 is very different in the provinces of Iran and its diversity depends on socioeconomic status, urbanization rate, and cultural and geographical issues of the provinces. Therefore, national estimation of exposure in the GBD Study precluded the authors from considering the distribution of exposure at provincial or regional level. 

Currently, a national systematic study, entitled “The National and Sub-national Burden of Disease (NASBOD) Study” is underway in Iran to estimate the burden of diseases, injuries, and risk factors at national and sub-national levels from 1990 to 2013.^30^ The NASBOD Study would provide comprehensive information to estimate the health status at provincial level for a long period of time. The sub-national analysis would be the major source for public health priorities and interventions as well as for planning public health strategies. 

## Conclusion

Although a declining trend in the burden of IHD attributable to a low omega-3 fatty acids intake in a period of 20 years was found according to the results of the GBD Study 2010, the attributable burden of total DALYs increased in 2010 compared to 1990 in Iran. Further disease burden studies at national and sub-national level in Iran drawing upon more data sources are suggested for public health priorities and interventions as well as for planning public health strategies.

## Notes:

***This paper should be cited as:*** Nejatinamini S, Ataie-Jafari A, Ghasemian A, Kelishadi R, Khajavi A, Kasaeian A, Djalalinia S, Saqib F, Majidi S, Abdolmaleki R, Hosseini M, Asayesh H, Qorbani M. Burden of Ischemic Heart Disease Attributable to Low Omega-3 Fatty Acids Intake in Iran: Findings from The Global Burden of Disease Study 2010. J Teh Univ Heart Ctr 2016;11(1):21-29.
